# Functional analyses of a novel missense and other mutations of the vitamin D receptor in association with alopecia

**DOI:** 10.1038/s41598-017-05081-x

**Published:** 2017-07-11

**Authors:** Mayuko Tamura, Michiyasu Ishizawa, Tsuyoshi Isojima, Samim Özen, Akira Oka, Makoto Makishima, Sachiko Kitanaka

**Affiliations:** 1 Department of Pediatrics, Graduate School of Medicine, The University of Tokyo, Tokyo, Japan; 2 The Japan Society for the Promotion of Science, Tokyo, Japan; 3 Division of Biochemistry, Department of Biochemical Sciences, Nihon University School of Medicine, Tokyo, Japan; 4 Department of Pediatric Endocrinology, School of Medicine, Ege University, Izmir, Turkey

## Abstract

Hereditary 1,25-dihydroxyvitamin D-resistant rickets (HVDRR) is a rare disorder, caused by bialellic mutations of the vitamin D receptor (VDR) gene, sometimes associated with alopecia. The aim of this study is to elucidate the mechanism of functional disruption of a novel mutation, detected in a patient with HVDRR, comparing to other mutations with or without alopecia. The patient was a 2-year-old girl with alopecia, who was clinically diagnosed as HVDRR. Genetic analysis revealed a novel homozygous mutation, S360P, located in ligand binding domain (LBD). The mutation was predicted as not disease causing by Polyphen2 and SIFT. But the transcriptional activity of S360P was disrupted as well as other reported mutations, Q152X (located in the hinge lesion), and R274L, H305Q (located in LBD). Following assays revealed no ligand binding affinity, no interaction with cofactors or RXR and no functioning of nuclear localization signals. Our results provide an additional evidence for the previous findings suggesting that DNA binding by the VDR/RXR heterodimer is essential for the function of the VDR in hair development. In conclusion, we identified a novel missense mutation of VDR causing HVDRR with alopecia. Functional analyses revealed that the single amino acid substitution could disrupt the function of the protein.

## Introduction

Hereditary 1,25-dihydroxyvitamin D (1,25[OH]_2_D)-resistant rickets (HVDRR) (OMIM #277440) is a rare disorder caused by homozygous mutations in the vitamin D receptor (VDR) gene. It is characterized by rickets symptoms, and sometimes alopecia^1^. Biochemical investigation of the blood shows hypocalcemia, hyperparathyroidism, and high levels of serum 1,25(OH)_2_D. Patients with HVDRR are resistant to 1,25(OH)_2_D_3_ and 1α(OH)D_3_ treatment. Elevated 1,25(OH)_2_D levels differentiate HVDRR from 1α-hydroxylase deficiency, known as vitamin D-dependent rickets type 1A^2^. The VDR is a member of the steroid/nuclear receptor superfamily of ligand-activated transcription factors, composed of an N-terminal DNA-binding domain (DBD) and a C-terminal ligand-binding domain (LBD)^3^. The VDR has other functional domains that contribute to the binding of cofactors, retinoid X receptor (RXR) heterodimerization, and nuclear localization signals (NLS)^4^. When acting as a transactivator, the VDR binds to a ligand, enters the nucleus, makes a heterodimer with the RXR, binds to vitamin D response elements, and transactivates target genes, while being modulated by cofactors. If any of these steps is disrupted, HVDRR occurs. Almost 50 mutations of the *VDR* gene have been reported^5^. Some mutations cause rickets symptoms, and alopecia^6^. Most mutations located on the DBD result in alopecia and severe rickets, whereas mutations on the LBD result in various degrees of rickets symptoms, with or without alopecia. Functional analyses of several LBD mutants associated with alopecia show an inability of the VDR to interact with the RXR, but the mechanism by which LBD mutants cause alopecia is not well understood. Comparing the functional features of mutants with and without alopecia would increase our understanding of the mechanism of hair development.

Several softwares are available for *in silico* studies to predict the functional impairment resulting from single amino acid substitutions. However, functional predictions with such software does not always reflect the actual effect of missense mutations^7–9^. In such cases, only functional analyses can provide the correct information. Additionally, comparing a substitution with other mutations in several functional studies provides more information about the substitution. It is intriguing to study how one amino acid substitution located on a certain functional domain impacts the whole function of the protein. Herein, we identified a novel missense mutation located on the LBD of the VDR in a patient with severe HVDRR with alopecia. We performed several functional analyses to investigate the effects on the whole VDR protein of the single amino acid substitution on the LBD, comparing its effects with those of some other mutations reported in patients with HVDRR. We found that the novel mutation disrupted the protein function, which finding was not predicted by the *in silico* analyses.

## Materials and Methods

### Patient

**Figure 1:**
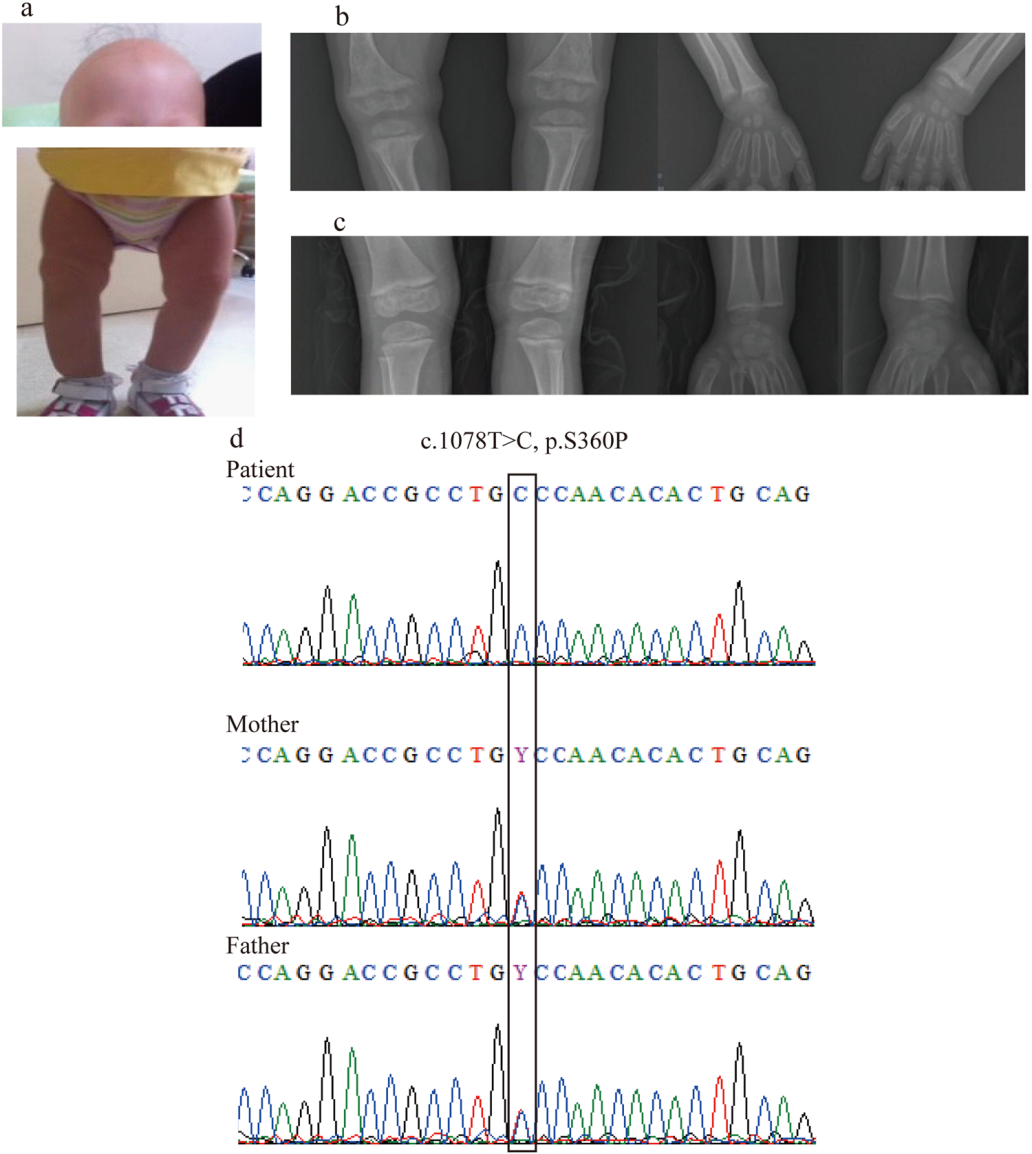
Clinical appearance of the patient and results of VDR gene analysis. (**a**) Pictures of the patient. The patient had almost total alopecia and bowlegs. An X-ray examination of the patient’s legs (**b**) before and (**c**) after 1 year of cinacalcet treatment. (**d**) Results of VDR gene analysis. The patient had a homozygous mutation, c.1078 T > C, p.S360P. Both parents were carriers.

We obtained informed consent of the patient’s parents for publication of identifying information and images. The patient was a 2-year-old Turkish girl admitted for total alopecia and bilateral bowlegs. Her weight was 9.2 kg (−2.1 SD) and her height 78.5 cm (−1.3 SD). She had total alopecia, frontal bossing, prominent costochondral junctions, widening of the wrists, and bowlegs (Fig. 1a). Alopecia began when she was 3 months old. Motor and mental development was normal. She was taking 400 IU of oral vitamin D daily. She was the first offspring of consanguineous parents. Laboratory examination revealed serum calcium 2.1 mmol/L (normal range, 2.1–2.5), serum phosphorus 0.77 mmol/L (normal range,0.97–1.78), alkaline phosphatase 1400 IU/L (normal range, 100–350), PTH 73.4 pmol/L (normal range, 1.3–6.4), 1,25(OH)_2_D 258 pmol/L (normal range, 39–102), and 25(OH)D 80 nmol/L (normal range, 50–250). X-rays of the left hand and wrist and the lower extremities were consistent with severe rickets (Fig. 1b). We diagnosed HVDRR clinically because of the rickets symptoms and alopecia.

We began treatment with calcitriol 6 µg/day, elementary calcium 150 mg/kg/day, and phosphate 100 mg/kg/day. After 1 year, we began cinacalcet treatment with 6 mg/kg/dose twice a day orally. While tapering and ceasing calcitriol and phosphate, we continued cinacalcet and elementary calcium. This regimen improved the rickets symptoms (Fig. 1c), but the alopecia remained.

### *VDR* gene analysis

We obtained written informed consent for DNA analysis from the parents. All the experiments of this study were performed in accordance with the institutional research guidelines and regulations, and approved by Ethics Committee of the University of Tokyo, Graduate School of Medicine and Faculty of Medicine. Genomic DNA was extracted from peripheral white blood cells of the patient and her parents with a QIAamp DNA Blood Midi Kit (Qiagen, Germany). The entire coding region and the exon–intron boundaries of the *VDR* gene were amplified from the genomic DNA by PCR using the specific primers previously reported^10^. The PCR products were subsequently sequenced in both directions by outsourcing (Eurofins Genomics, Japan).

### Construction of plasmids

The constructs of wild-type human *VDR* expression plasmids pCMX-flag-hVDR, pCMX-VP16-hVDR, pCMX-GAL4-RXRα, pCMX-GAL4-SRC-1, and pCMX-GAL4-N-CoR were previously reported^11^. VDR-responsive SPPx3-tk-LUC and GAL4-responsive MH100(UAS)x4-tk-LUC were also used in the luciferase reporter assay^12^. Mutations of *VDR* were introduced into cloning vectors by site-directed mutagenesis and cloned in pCMX-flag-hVDR, pCMX-VP16-hVDR, and pCMX-GFP-hVDR. *VDR* mutant constructs were sequence-verified to have no extra mutations. Translation of constructed vectors to proteins was confirmed by Western blotting, in which proteins were collected after 24 hours of transfection.

### Cell culture and transfection

Human embryonic kidney HEK293 cells (RIKEN Cell Bank, Japan) and monkey kidney epithelial cell line COS-1 cells were cultured in DMEM containing 5% FBS, 100 U/mL penicillin, and 100 μg/mL streptomycin at 37 °C in a humidified atmosphere containing 5% CO_2_. Transfections in HEK293 cells were performed by the calcium phosphate coprecipitation method, as previously described^13^. Transfections in COS-1 cells were performed by modification of previously published methods^14^, using Lipofectamine 2000 (Invitrogen, Carlsbad, USA).

### Luciferase assay and mammalian two-hybrid assay

DNA cotransfection experiments were performed with HEK293 cells, including 50 ng of reporter plasmid, 10 ng of pCMX-β-galactosidase, and 15 ng of each expression plasmid for each well of a 96-well plate. 8 hours after transfection, ligand was added. The cells were harvested approximately 16–24 hours after the addition of ligand, and luciferase and β-galactosidase activities were assayed with a luminometer and microplate reader (Molecular Devices, Sunnyvale, USA). Luciferase data were normalized to an internal β-galactosidase control and represent the means (±SD) of triplicate assays.

### Ligand-binding assay

The pGEX, pGEX-hVDR, and its mutants were expressed as a GST fusion protein in *Escherichia coli* BL21 DE3 (EMD Millipore, USA). Using GST-VDR fusion protein induced by *Escherichia coli* BL21 DE3, a ligand-binding assay was performed by modification of previously published methods^15^. Briefly, 1 µg of GST fusion proteins was incubated with [^3^H]1α,25(OH)_2_D_3_ (PerkinElmer, USA) for 20 hours at 4 °C. After mixing with dextran-coated charcoal (DCC) and centrifuging, a liquid scintillation cocktail was added to the supernatant. Liquid scintillation counting was used to assess the mixture.

### Fluorescence microscopy

COS-1 cells cultured in Collagen Type I-Coated Chamber slide (IWAKI, Japan) were transfected with 800 ng of pCMX-GFP-VDR including VDR mutants. After 24 hours, 100 nM of ligand, 1,25(OH)_2_D_3_, or vehicle (ethanol) was added. After 4 hours of incubation, 10 ng/mL of Hoechst (Invitrogen, USA) was added to each chamber for staining of the nucleus, and then microscopy was performed with an inverted microscope (model IX71; Olympus, Japan). Images were obtained with a cooled charge-coupled device camera (ORCA-ER; Hamamatsu Photonics, Japan) controlled by Aqua-Lite software (Hamamatsu Photonics, Japan). For quantitative analysis, images were processed by ImageJ^16^. 20 cells in images of each mutant were randomly selected, and the mean brightness of the nuclei defined by Hoechst images was compared with the mean brightness of the cytoplasm for each cell. The average data for each mutant were statistically analyzed using unpaired Student’s *t*-test.

## Results

### *VDR* gene analysis

*VDR* gene analysis revealed a homozygous substitution from serine to proline at position 360 (c.1078 T > C) of the VDR in the patient, and the parents were carriers (Fig. 1d). This mutation, located in the LBD, has not been described as a mutation of *VDR*. It is not found in the single nucleotide polymorphism (SNP) database of the 1,000 Genome Project^17^ or in house databases of Japanese SNPs. Analysis *in silico* by Polyphen2^18^ and SIFT^19^ yielded “benign” and “tolerated” results, respectively.

### Transcriptional activity

**Figure 2:**
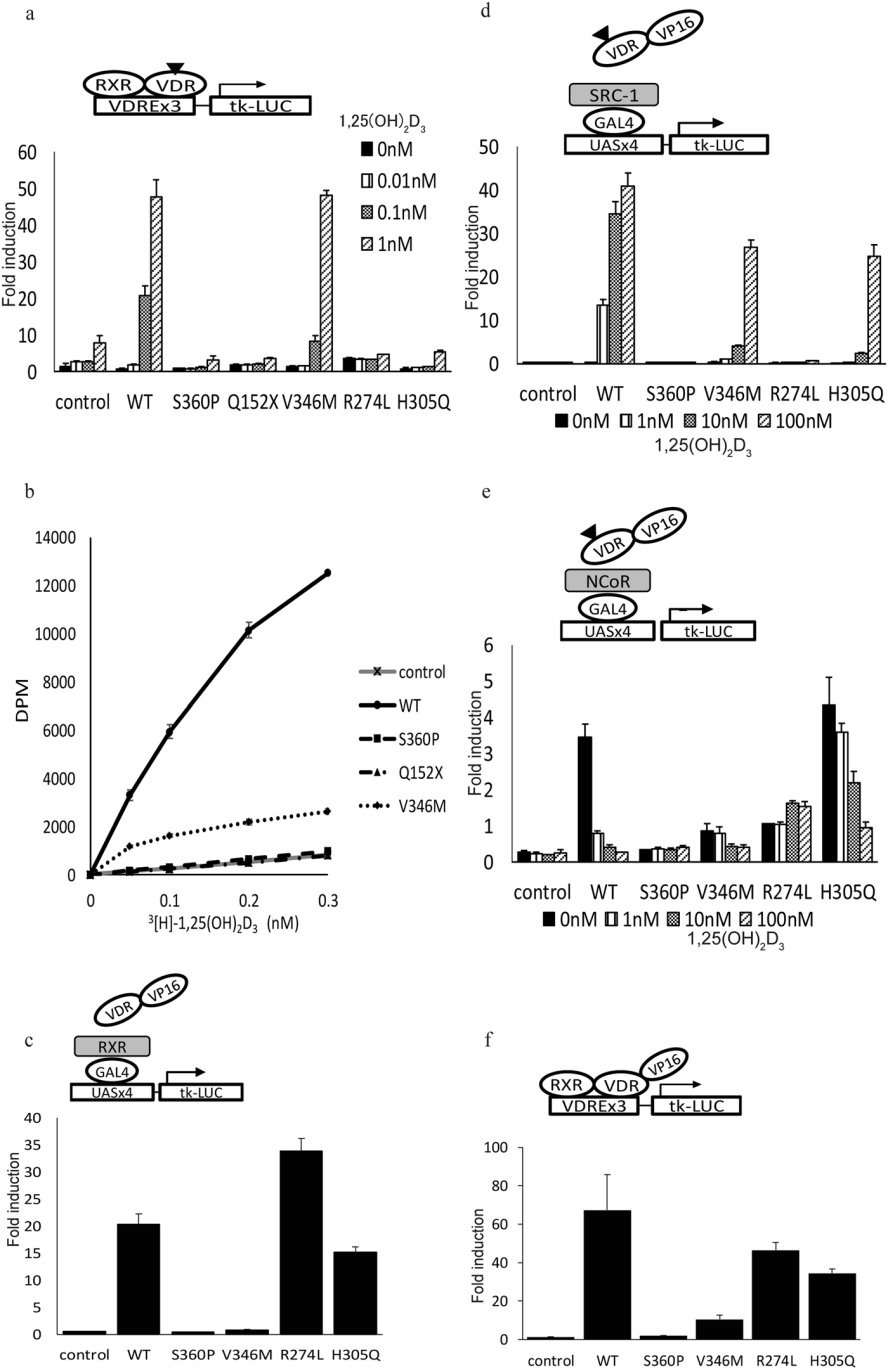
VDR functional analyses: Transcriptional activity, ligand-binding affinity, RXR interaction, cofactor interactions, and enhanced transcriptional activity. (**a**) Transcriptional activities of VDR wild type and mutants. VDR expression vectors or a control vector and a SPPx3-tk-LUC reporter were transfected into HEK293 cells and treated with 1α,25(OH)_2_D_3_. Each mutant exhibited relatively low activity than the wild type. The transcriptional activity of V346M was improved with high doses of the ligand. (**b**) Ligand-binding affinity of the VDR. S360P had no affinity, showing almost the same level as control and Q152X, with no ligand-binding domain. V346M had little affinity compared with control. (**c**) Interaction between the VDR and RXRα in the absence of ligand. S360P and V346M showed no interaction with the RXRα compared with R274L. H305Q and the wild type showed interaction. (**d**) Interaction between the VDR and SRC-1 determined by a mammalian two-hybrid assay. S360P and R274L showed no interaction with SRC-1. (**e**) Interaction between the VDR and N-CoR. Wild-type VDR revealed a high level of interaction with N-CoR with no ligand and less interaction with increasing amounts of the ligand. S360P showed no interaction with N-CoR, even without the ligand. (**f**) Enhanced transcriptional activity, which precedes DNA binding since the activity proceeds even without ligand binding. Wild type, R274L, and H305Q showed built-up activity, whereas S360P and V346M did not. Those mammalian two-hybrid analysis and the enhanced transcriptional activity assay were performed transfecting vectors into HEK293 cells. n = 3 biological replicates; data represent means ± SD.

To investigate the ability of *VDR* mutants to activate transcription of target genes, we performed a transcriptional activity assay with a luciferase assay using several *VDR* mutants in addition to S360P. V346M is a representative missense mutation in the LBD found in patients with alopecia^20^. R274L and H305Q are representative missense mutations in the LBD in patients without alopecia^21, 22^. Q152X is a nonsense mutation on the hinge of the VDR, missing the LBD, which is reported to cause severe rickets with alopecia^21^. As a result, control showed ligand dependent luciferase activity slightly because of endogenous VDR of HEK293 cells as previously reported^23^. The activity was effectively induced by expression of the wild type VDR, on the other hand, transcriptional activities were impaired in the mutants S360P, Q152X, V346M, R274L, and H305Q (Fig. 2a). This result indicated that S360P and the other mutations are disease-causing mutations. This was the first functional analysis of V346M, and it indicated that V346M is also a disease-causing mutation. The activity of V346M was increased with high-dose 1,25(OH)_2_D_3_ (Fig. 2a), a finding consistent with the improvement of rickets by alfacalcidol in one case^20^.

### Ligand-binding affinity

We further investigated how the mutant lost the ability to activate transcription. We first investigated ligand-binding affinity, which is critical for the function of the VDR in calcium homeostasis. Some mutations associated with negative ligand-binding properties cause HVDRR^24^. R274L and H305Q have been demonstrated to have no or little ligand-binding affinity^21, 22^. In our assay, we confirmed that the binding of control GST was background level and that the GST-VDR protein showed significant binding to [^3^H]1α,25(OH)_2_D_3_ (Fig. 2b). S360P had no ligand-binding affinity, almost the same as that of the control and Q152X, which is lacking the LBD (Fig. 2b). The ligand-binding affinity of V346M was partially impaired (Fig. 2b).

### Interaction with the RXRα

Heterdimerization with the RXR is also one of the crucial roles of the VDR in binding the vitamin D response element of the target genes and activating transcription. Some mutants reportedly have impaired RXR binding^25, 26^. Moreover, RXR heterodimerization has been suggested to be critical for the function of the VDR in hair development^6^. Some reports have demonstrated that the VDR function in the hair follicle does not require a ligand^27, 28^. Therefore, the result of interaction with the RXRα without ligand binding gives profitable information to understand each mutant with regard to occurring alopecia. In the absence of ligand, S360P, and V346M, which are associated with a phenotype with alopecia, showed no interaction with the RXRα. R274L and H305Q, which are not associated with alopecia, interacted with the RXRα to the same extent as did the wild type (Fig. 2c). With ligand adding, the interaction increases in a dose-dependent manner in the mutants that have ligand-binding affinity (V346M, R274L, and H305Q) (Supplementary Fig. S1a). The interaction of the V346M mutant with the RXRα was especially improved with the addition of a large amount of ligand (Supplementary Fig. S1a). S360P did not interact with the RXRα in the presence or in the absence of ligand (Fig. 2c, Supplementary Fig. S1a). These results indicate that the mutants associated with alopecia (S360P and V346M) did not interact with the RXRα, whereas the mutants not associated with alopecia (R274L and H305Q) did interact with the RXRα. In these mammalian two hybrid assays, the reporter activities are not affected by endogenous VDR.

### Interaction with cofactors

Next, we performed a cofactor interaction assay by mammalian two-hybrid assay. Decreasing interactions with coactivators or increasing interactions with corepressors cause disruption of VDR function and result in HVDRR^28^. Interaction with steroid receptor coactivator-1 (SRC-1) was seen with V346M and H305Q, even though it was smaller than that with the wild type, but not with S360P and R274L (Fig. 2d), which was consistent with the fact that ligand binding was necessary to increase interaction for coactivators. On the other hand, ligand binding reduces interaction for corepressors. Consistent with this, wild-type VDR interacts with the corepressor N-CoR without a ligand and decreases the interaction in a dose-dependent manner (Fig. 2e). H305Q interacted even more than the wild type, V346M and R274L had less interaction, and S360P had no interaction with N-CoR, even without a ligand (Fig. 2e). The results showed that S360P could not interact with SRC-1 or with N-CoR.

### Enhanced transcriptional activity

To presume DNA-binding ability, we performed an enhanced transcriptional activity assay using VDR-VP16 fusion protein. In this assay, transcriptional activity builds up and proceeds even without ligand binding. Thus, this assay detects presumed DNA binding^29^. As expected, transcriptional activity of the wild-type fusion protein was built up (Fig. 2f). The transcriptional activities of R274L and H305Q were also built up compared with normal transcriptional activity, in the absence of ligand (Fig. 2f). However, the fusion protein of S360P had no transcriptional activity, with or without ligand (Fig. 2f, Supplementary Fig. S1b). Although this assay depended on binding to endogenous RXR, in consequence S360P was presumed to have defective DNA-binding ability.

### Intracellular localization

**Figure 3:**
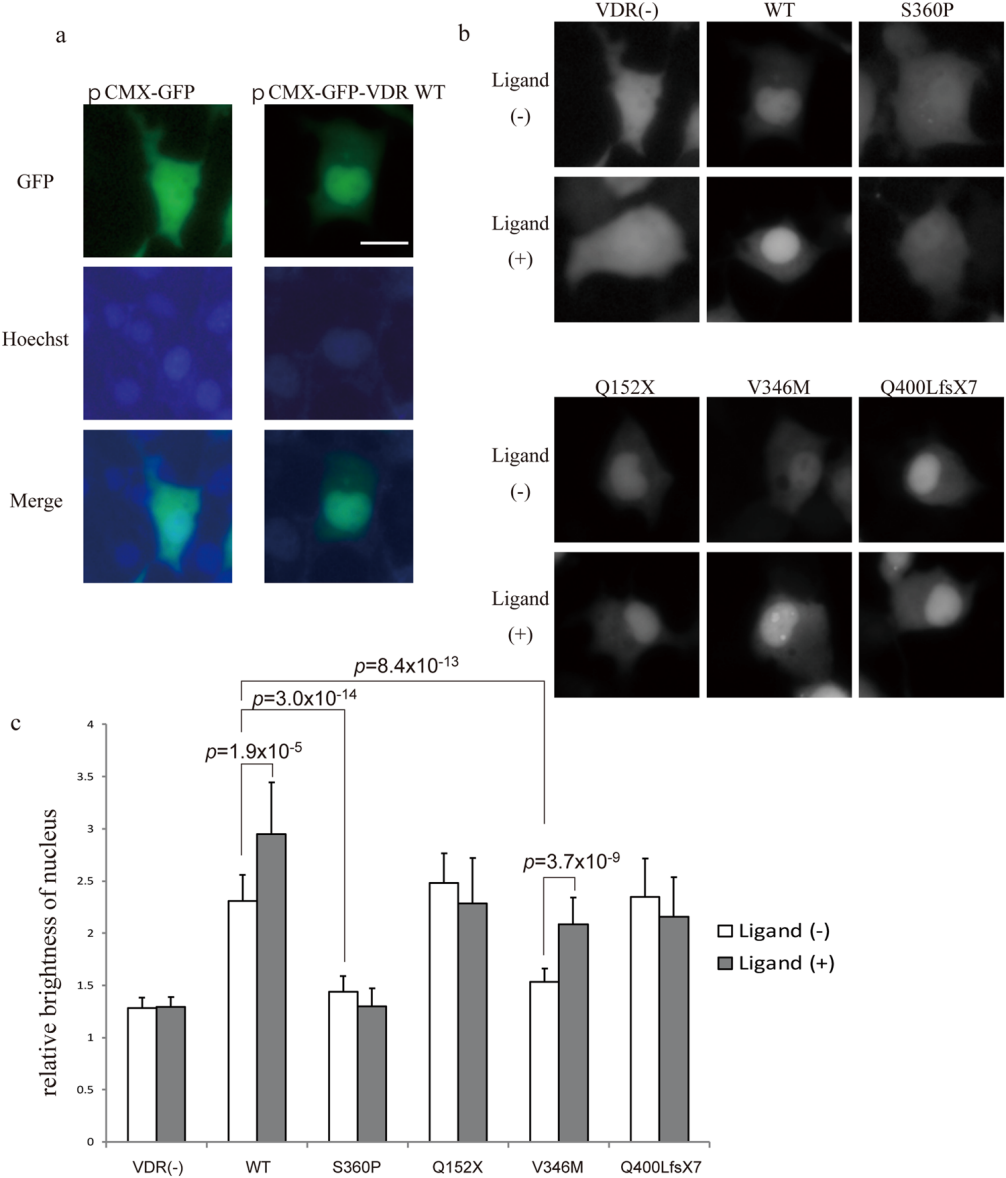
Intracellular localization of wild-type and mutant VDR. (**a**) Representative image of a COS-1 cell transfected with pCMX-GFP and pCMX-GFP-VDR wild type. GFP is shown in green and Hoechst in blue. pCMX-GFP is distributed in the whole cell, and wild-type VDR is partially localized in the nucleus. Scale bar: 20 µm (**b**) Representative GFP images of a COS-1 cell transfected with pCMX-GFP, -VDR wild type, and each mutant with and without the ligand. (**c**) The ratio of mean brightness of nucleus and cytoplasm analyzed by Image J. n = 20. Data represent means ± SD. Unpaired two-tailed Student’s *t*-test.

The VDR needs to enter the nucleus for transactivation, which is performed by nuclear localization signals (NLS). Fluorescence microscopy of GFP-VDR transfected to COS-1 cells gives information about protein translocation in cells. In this assay, we used wild-type VDR and S360P, Q152X, and V346M mutants. In addition, we used Q400LfsX7, a frameshift mutation with a dominant negative effect and no ligand-binding affinity, as we reported previously^29^. The results showed that the fusion protein consisting of GFP and wild-type VDR was localized mainly in the nucleus, even without ligand, and that localization was enhanced with ligand, which is consistent with results reported previously^30^, whereas GFP alone was distributed over the whole cell (Fig. 3a). We defined a nucleus lesion by merging images stained by Hoechst (Fig. 3a). Among VDR mutants, S360P was localized very weakly in the nucleus before ligand binding, and localization was not enhanced after ligand adding (Fig. 3b). V346M was also less localized in the nucleus, but its localization was better than that of S360P and was enhanced with the addition of ligand (Fig. 3b). Other mutants, as well as the wild type, were localized much more in the nucleus, but localization was not enhanced in the mutants by adding ligand, because they had no ligand-binding affinity (Fig. 3b). Quantified analysis of the images was performed by ImageJ. The mean brightness values of the nucleus and cytoplasm were measured. Figure 3c shows the ratio of brightness values of the nucleus to those of the cytoplasm. This intracellular localization assay showed disrupted nuclear localization in S360P.

## Discussion

**Figure 4:**
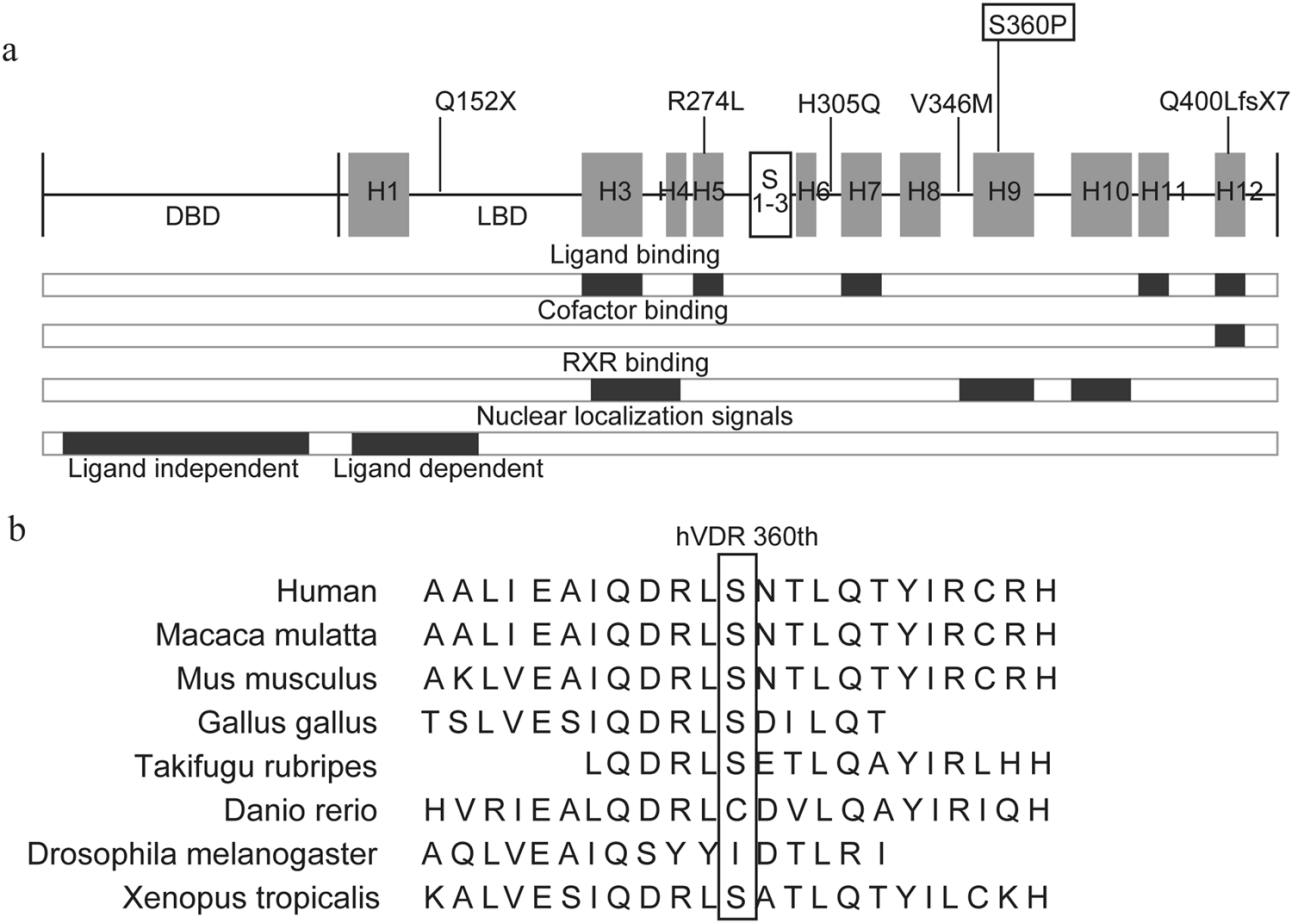
Functional domains of the VDR protein and homologues of the 360^th^ residue. (**a**) Functional domain chart of the VDR protein. Gray boxes represent helices of the ligand-binding domain (LBD). Each mutant is exhibited on each location; Q152X, which is a nonsense mutation on the hinge of the VDR, missing the LBD^21^, R274L and H305Q, missense mutations in the LBD found in patients without alopecia^21, 22^, V346M, a missense mutation in the LBD found in patients with alopecia^20^, Q400LfsX7, a frameshift mutation without alopecia^29^, and S360P, the novel missense mutation we reported in this study. Bars below the protein chart show functional domains based on previous reports of ligand binding, cofactor binding, RXR binding, and nuclear localization signals^4, 31^. (**b**) Homologs of the 360^th^ residue of the VDR among species. Serine, the 360^th^ residue of human VDR, is not a highly conserved residue.

This is the first reported case of a patient with a mutation in the 360th residue of the human VDR, located on helix 9 of the LBD (Fig. 4a), which was associated with rickets and alopecia. *In silico* studies by Polyphen2 and SIFT predicted that the substitution in S360P would not disrupt the function of the protein. Moreover, the residue is not highly conserved in other vertebrates (Fig. 4b). Nevertheless, the clinical features of severe rickets unresponsive to high-dose vitamin D therapy, combined with alopecia, indicated a severe defect in the function of the VDR.

To investigate the discrepancy between the *in silico* studies and the clinical features, and to confirm the molecular cause of this patient’s clinical features, we performed *in vitro* analysis of the mutant. Analysis revealed the disrupted transcriptional activity of S360P, demonstrating that the mutation caused HVDRR. To further investigate the mechanism of disruption of VDR function, we performed several functional analyses, comparing the findings with those from wild-type VDR and other reported mutants. First, the ligand-binding affinity assay showed that S360P had no ligand-binding affinity. The ligand-binding pocket is bordered by helices H3, H5, H7, and H11 and loop-encompassing residues that include S275 (loop H5-β), W286 (β-1), and L233 (H3), with a lid formed by H12; residues H305 and H397 also play a role in the ligand-binding pocket^4, 31^. Considering that S360P does not appear to affect ligand binding directly (Fig. 4a), it might have a distinctive mechanism to disrupt VDR function. Second, RXR and cofactor interactions were impaired in S360P. S360 belongs to helix 9, which has an important role in RXR binding (Fig. 4a). This mutant lost the ability to interact with the RXR. On the other hand, S360P (H9) is not located in the region contributing to binding with cofactors (Fig. 4a). S360P did not interact with SRC-1, which was supposed to be because interaction with SRC-1 depends on ligand binding and S360P lacks ligand-binding affinity. Conversely, N-CoR interaction should present highest degree before ligand binding, but interaction was not seen in S360P. Because S360P retains the region responding the LXXLL motif of cofactors, these findings suggest that S360P disrupts the VDR structure more than would be expected from a single amino acid substitution.

Herein, we performed several functional analyses comparing S360 with other mutants, to understand the distinctive S360P features. This is the first functional analysis demonstrating that V346M is also a mutation causing HVDRR. The transcriptional activity of V346M was improved with high doses of ligand, and ligand-binding affinity partially remained, consistent with the clinical course described previously^20^.

The intracellular localization study provided further information about the mutants. The S360P mutant presented a different pattern from that of the wild type and of other mutants, showing very weak localization in the nucleus, which was not enhanced in the presence of ligand. V346M also showed weak localization in the absence of ligand, but localization was improved with ligand addition. The VDR has two types of NLS. One is dependent on ligand binding and is located in the hinge region; the other is independent of ligand and is located in the DBD (Fig. 4a)^32^. Our data suggest that NLS independent of ligand binding were impaired in the S360P and V346M mutants, but that NLS dependent on ligand binding works in the V346M mutant, suggesting that S360P and V346M, which are both single amino acid substitutions on the LBD, might be detrimental to NLS function in the DBD. It is clear that NLS independent of ligand binding works in Q152X, and Q400LfsX7, but NLS dependent on ligand binding does not work in either of these mutants, which have been demonstrated not to have ligand-binding affinity.

Alopecia is a distinctive sign of HVDRR. Previous reports suggested that alopecia occurred in mutants that could not form a heterodimer with the RXR or that had a defect in DNA binding, whereas ligand-binding ability was unrelated^27, 33^. Thus, we considered that whether a mutant can interact with the RXR in the absence of ligand is important with regard to alopecia. Recently, the V26M mutant in a patient with alopecia reportedly indicated that DNA-binding ability and not RXR heterodimerization is crucial^34^. Herein, we showed that mutant VDR in patients without alopecia (R274L and H305Q) interacted with the RXR and had presumed DNA binding, but mutant VDR in patients with alopecia (S360P and V346M) did not interact with the RXR or have presumed DNA binding in the absence of ligand. These results were consistent with previous studies and we also think that the DNA-binding ability of the VDR/RXR heterodimer is essential for the function of VDR in hair development, and that DBD mutants lacking DNA-binding ability and LBD mutants lacking RXR-binding ability may result in a DNA-binding defect^6, 33^. The fact that the transcriptional activity and RXR interaction of V346M were improved by the addition of ligand corresponds to the reported clinical course in which treatment with alfacalcidol improved rickets symptoms but alopecia remained^20^, indicating that alopecia may occur independently of ligand binding. The Hairless gene (*HR*) is the other key factor in hair follicle cycles, and *HR* mutation results in hair-loss phenotypes similar to those associated with VDR mutations^35^. Some mutants of VDR were reportedly less suppressed by *HR*, suggesting the interface of *HR*, which interacts with the VDR^36^. Further research is necessary to elucidate the mechanism of alopecia in HVDRR and possible treatments for hair loss.

The impaired VDR function of S360P identified in this study is consistent with the patient’s clinical presentation as severe HVDRR with alopecia. According to the chart of VDR functional domains, the 360^th^ residue does not seem to contribute to any of these functions, except for RXR binding (Fig. 4a). However, a single amino acid substitution of serine to proline causes extensive disruption of the function of the VDR protein. This possibly occurs because proline, the only cyclic amine, breaks helix 9 with damage to the whole protein structure, leading to all the defects of ligand binding, cofactor interaction, RXR heterodimerization, and localization in the nucleus.

The limitation of this study is that the assays were performed with overexpression of fusion proteins although protein expression after 24 hours of transfection was confirmed by western blot. It also harbored the possibility that the instability of the mutant proteins influenced the result of overexpression assay. Ideally, we should analyze the function of S360P in the patient’s tissue such as skin fibroblasts, although we could not obtain fibroblasts from the patient. Reporter assay is also one of significant assays, which reflect specific characters of the mutation. There are quite a few studies, in which only reporter assays were performed to investigate functional defects, not only for VDR^21, 37^ but also for other nuclear receptors, such as androgen receptor^38^, and thyroid hormone receptor β^39^. Moreover, our results showing considerable differences among the mutants suggest that those are replicating the clinical severity and the ineffectiveness to calcitriol treatment.

In this study, *in silico* models of S360P did not match the functional analyses, and this kind of inconsistent predictions could happen reportedly. In particular, Polyphen2, which integrates information including protein structure, could not detect such a vast disruption throughout the function of the whole protein. Each software has its own accuracy, and the accuracy of predictions for specific genes and diseases varies^40^, indicating that undiscovered mechanisms for the disruption of protein function with missense mutations still exist. In this case, we suggest as one of the reasons for the unpredicted results, that serine, the 360th residue, is not a highly conserved residue. There is no objection to using computer software broadly to predict the functional effects of a mutation; however, we should consider the possibility of misleading results from an *in silico* study, and we consider that functional analyses will be necessary to confirm the functional consequences of a mutation.

## Conclusions

We identified a novel missense mutation of the *VDR* gene that causes HVDRR with alopecia. Functional analyses revealed various features of several mutations of *VDR* in patients with or without alopecia, and showed that a single amino acid substitution could cause wide disruption of the protein function.

## Electronic supplementary material

Supplementary Information.
