# Supplementary material for: Functional analyses of a novel missense and other mutations of the vitamin D receptor in association with alopecia
Source: Sci Rep. 2017 Jul 11;7:5102. doi: 10.1038/s41598-017-05081-x (PMC5505967; doi:10.1038/s41598-017-05081-x)
Supplement: Supplementary file 1 — Supplementary Information. [file 41598_2017_5081_MOESM1_ESM.pdf]

Supplementary Information

Functional analyses of a novel missense and other mutations of the vitamin D receptor in association with alopecia

Mayuko Tamura, Michiyasu Ishizawa, Tsuyoshi Isojima, Samim Özen, Akira Oka, Makoto Makishima, Sachiko Kitanaka

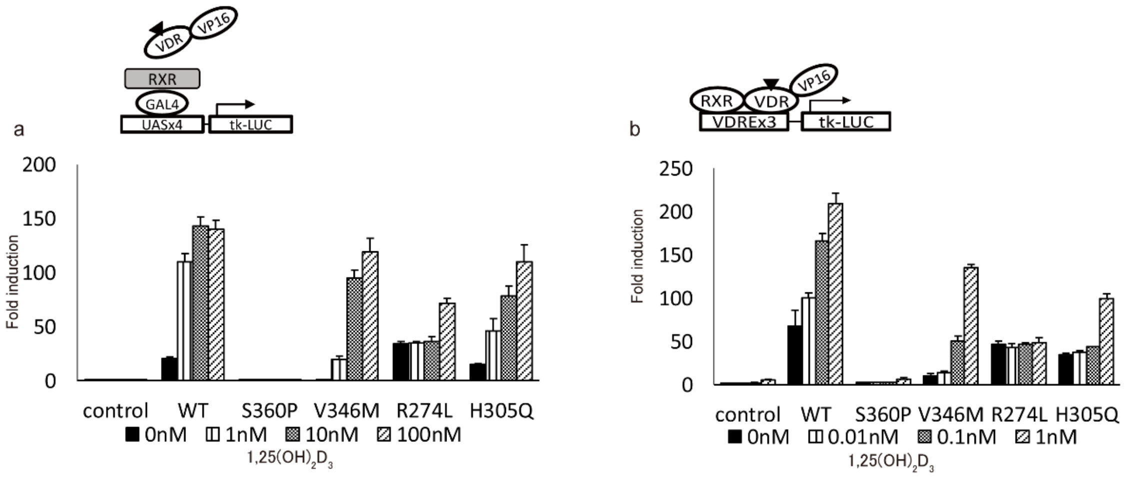

Supplementary Figure S1. Interaction with RXRα and enhanced transcriptional activity in the absence and presence of ligand.

(a) Although V346M showed very week RXRα interaction in the absence of ligand, it was increased in dose dependent manner of ligand. S360P didn't change before and after ligand adding. (b) Enhanced transcriptional activity showed built-up activity in the presence of ligand in mutants which had ability of ligand binding. n=3 biological replicates; data represent mean ±S.D.
